# Effects of microenvironment and biological behavior on the paracrine function of stem cells

**DOI:** 10.1016/j.gendis.2023.03.013

**Published:** 2023-04-11

**Authors:** Zhixin Xue, Yunjun Liao, Ye Li

**Affiliations:** The Department of Plastic and Cosmetic Surgery, Nanfang Hospital, Southern Medical University, Guangzhou, Guangdong 510515, China

**Keywords:** Bioactive behavior, Mesenchymal stem cells, Microenvironment, Paracrine, Secretome

## Abstract

Mesenchymal stem cells (MSCs), the most well-studied cell type in the field of stem cell therapy, have multi-lineage differentiation and self-renewal potential. MSC-based therapies have been used to treat diverse diseases because of their ability to potently repair tissue and locally restore function. An increasing body of evidence demonstrates that paracrine function is central to the effects of MSC-based therapy. Growth factors, cytokines, chemokines, extracellular matrix components, and extracellular vehicles all contribute to the beneficial effects of MSCs on tissue regeneration and repair. The paracrine substances secreted by MSCs change depending on the tissue microenvironment and biological behavior. In this review, we discuss the bioactive substances secreted by MSCs depending on the microenvironment and biological behavior and their regulatory mechanisms, which explain their potential to treat human diseases, to provide new ideas for further research and clinical cell-free therapy.

## Introduction

Stem cells are a unique group of cells defined by their ability to self-renew, differentiate into multiple cell lineages, and form clonal cell populations. These characteristics mean that stem cells have a great potential to repair tissue, and these cells have been widely utilized for clinical treatment. Many types of stem cells are being clinically researched, and they can be divided into four categories: embryonic stem cells, stem cells derived from the placenta or amniotic fluid, pluripotent stem cells reprogrammed using specific transcription factors, and adult stem cells. Mesenchymal stem cells (MSCs) are a type of adult stem cells that exist in bone marrow, muscle, and adipose tissue. They are the most well-studied cell type in the field of stem cell therapy and are mainly involved in tissue repair and local function restoration. Meanwhile, MSCs can elicit immunoregulatory effects and are therefore a hot topic in the field of regenerative medicine, and the administration of MSCs holds great promise to treat diseases. However, the therapeutic potential of MSCs for clinical applications is limited by a series of shortcomings such as the decreased homing ability, low survival rate, and impaired differentiation ability of transplanted MSCs.

An increasing body of evidence demonstrates that paracrine function is central to the effects of MSC-based therapy. MSCs can regulate tissue regeneration and repair by releasing a variety of paracrine functional molecules such as growth factors, inflammatory cytokines, chemokines, and extracellular matrix (ECM) components. Some clinical trials have demonstrated the potential of conditioned medium of MSCs (MSC-CM) to treat a variety of diseases. For example, treatment with the MSC-derived product exosome-derived multiple allogeneic protein paracrine signaling (Exo-d-MAPPS) significantly improved the pulmonary status and quality of life of chronic obstructive pulmonary disease patients^1^; treatment with 5% MSC-CM improved androgenetic alopecia by increasing hair density, hair thickness, and the hair growth rate^2^; and the skin barrier was strengthened due to decreased transepidermal water loss following application of MSC-CM to lesions of patients with mild atopic dermatitis for 4 weeks.^3^ Considering that the paracrine functions of MSCs play an important role in disease therapy, changes of the paracrine substances secreted by these cells according to the microenvironment and biological behavior have been studied. In addition, ways to regulate the physiological state and tissue microenvironment of MSCs and thereby change their secretion profile have begun to be researched to improve their therapeutic effects.

This article reviews the bioactive substances secreted by MSCs according to their microenvironment (Table 1) and biological behavior (Table 2) and regulatory mechanisms. It aims to provide new ideas for further research and clinical cell-free therapy.

**Table 1:** The paracrine effects of MSCs in different microenvironments.

Model	Microenvironment	Paracrine substance(s) released by MSCs	Effect	Reference
IBD mouse model	Hypoxia	IGF-2	Anti-inflammation	Fang et al^9^
MI mouse model	Hypoxia	VEGF	Angiogenesis	Gao et al^10^
Rat alveolar injury model	Hypoxia	KGF and HGF	Anti-inflammation and anti-fibrosis	Bernard et al^11^
ALI mouse model	Hypoxia	MSCs-exosomal miR-30b-3p	Inhibition of apoptosis of alveolar epithelial cells	Yi et al^12^
Lung ischemia/reperfusion mouse model	Hypoxia	MSCs-exosomal miR-21-5p	Inhibition of apoptosis of alveolar epithelial cells and alleviation of ischemia-reperfusion injury in the lung	Li et al^13^
Mouse bone fracture model	Hypoxia	MSCs-exosomal miR-126	Proliferation and migration of endothelial cells	Liu et al^14^
Mouse full-thickness skin injury model	Hypoxia	MSCs-exosomal miR-125b	Angiogenesis, proliferation, and migration of endothelial cells	Zhang et al^15^
Rat OA model	Hypoxia	BMSC-derived EVs containing miR-216a-5p	Stimulation of proliferation and migration and inhibition of apoptosis of chondrocytes	Rong et al^16^
Mouse ARDS model	Hypoxia	MSC-derived EVs containing mitochondria	Anti-inflammation: transformation of macrophages to the M2 phenotype	Morrison et al^17^
Mouse lipopolysaccharide-induced ALI model	Hypoxia	MSC-derived EVs containing Ang-1 mRNA	Immunomodulation of macrophages	Tang et al^18^
Culture of hMSCs	Hypoxia	IL-1β	Pro-inflammation and immune escape: inhibition of T cell proliferation	Miranda et al^35^
Culture of hypox-visASCs from patients with metabolic syndrome	IL-1β	IL-4, IL-10, IL-8, IL-13, FGF-2, EGF, VEGF-A, and MCP-1	Immunoregulation and angiogenesis	Oliva et al^19^
Mouse skin wound model	IFN-γ and TNF-α	VEGF-C	Angiogenesis of endothelial cells	Zhu et al^20^
Mouse cutaneous wound healing model	TNF-α	IL-6	Anti-inflammation: transformation of macrophages to the M2 phenotype	Liu et al^21^
Mouse prostate cancer growth model	IFN-γ and TNF-α in the TME	TGF-β1	Stimulation of invasion and inhibition of apoptosis of tumor cells	Jing et al^34^
Co-culture of prostate cancer cells and MSCs	Contact with tumor cells	TGF-β	Promotion of the EMT	Calon et al^22^
Co-culture of human BCCs and MSCs	Contact with BCCs	CCL5 and SDF-1α	Increase the metastatic potential of cancer cells	Karnoub et al^23^
Co-culture of osteosarcoma cells and BMSC-derived exosomes	Contact with osteosarcoma cells	BMSC-derived exosomes containing lncRNAPVT1	Proliferation, migration, and invasion of osteosarcoma cells	Zhao et al^24^
Mouse tumor xenograft model	Contact with lung cancer cells	MSC-derived EVs containing miR-130b-3p	Tumorigenesis of lung cancer	Guo et al^25^
Mouse tumor xenograft model	Contact with BCCs	BMSCs-exosomal miR-23b	Dormancy of BCCs	Ono et al^26^
Co-culture of human BCCs and BMSCs	Contact with BCCs	MSCs-exosomal miR-100	Inhibition of tumor growth and angiogenesis of endothelial cells	Pakravan et al^27^
Nude mouse model of tumor growth	Contact with lung cancer cells	BMSC-EV-derived let-7i	Inhibition of lung cancer	Liu et al^28^
Mouse lung tumor growth xenograft model	Lung tumor cell A549-derived exosomes	IL-6, IL-8, and MCP-1	Promotion of lung tumor growth	Li et al^29^
AML-engrafted mouse model	TEX produced by AML cells	CXCL12, KITL, IL-7, and IGF-1	Promotion of leukemic cell proliferation and survival, and suppression of normal hematopoiesis	Kumar et al^30^
Tumors co-injected with CSC-derived EV-stimulated MSCs in SCID mice	CSC-derived EVs	CXCR4, COL4A3, IL-8, osteopontin, and myeloperoxidase	Matrix remodeling, angiogenesis of endothelial cells, and migration and tumor growth of CSCs	Lindoso et al^31^
Osteosarcoma xenograft model in nude mice	Osteosarcoma-derived EVs	IL-6	Promotion of tumor growth	Baglio et al^32^
Co-culture of MSCs and tumor cell-derived EVs	Cholangiocarcinoma cell-derived EVs	IL-6	Proliferation of tumor cells	Haga et al^33^
An MSC culture system with shear stress for 4 h	Laminar shear	Cardiovascular support factors	Cardiovascular development and protection and angiogenesis of endothelial cells	Chen et al^36^
*In vitro* system mimicking the mechanical environment in the early bone healing phase	Cyclic compression	BMP	Osteogenic differentiation of MSCs	Schreivogel et al^37^
Hydrogel matrices with varying levels of stiffness	Matrix stiffness	VEGF	Angiogenesis of endothelial cells	Nasser et al^39^
MSCs cultured on the full N-cadherin extracellular domain	Cadherin	VEGF and IGF-1	Angiogenesis of endothelial cells	Qin et al^40^
Mouse MI model	Laminin	GRO-α, IL-8, and HGF	Inhibition of ROS production and cardiomyocyte apoptosis	Peng et al^41^

Abbreviations: ALI, acute lung injury; AML, acute myelocytic leukemia; ARDS, acute respiratory distress syndrome; BCCs, breast cancer cells; BMP, bone morphogenetic protein; BMSCs, bone marrow mesenchymal stem cells; COL4A3, collagen, type IV, alpha 3; CSCs, cancer stem cells; EGF, epidermal growth factor; FGF-2, fibroblast growth factor-2; GRO-α, human growth regulatory oncogene alpha protein; HGF, hepatocyte growth factor; IBD, inflammatory bowel disease; IGF-2, insulin-like growth factor-2; KGF, keratinocyte growth factor; KITL, kit ligand; MCP-1, monocyte chemoattractant protein-1; MI, myocardial infarction; OA, osteoarthritis; ROS, reactive oxygen species; SCID, severe combined immune deficiency; SDF-1α, stromal cell-derived factor-1α; TGF, transforming growth factor; VEGF, vascular endothelial growth factor.

**Table 2:** Paracrine effects of MSCs with different biological behaviors.

Model	Biological behavior of MSCs	Paracrine substance(s) released by MSCs	Effect	Reference
Co-culture of polarized macrophages and MSCs	Interaction between MSCs and M1 macrophages	CD54, CD200, PGE2, TSG-6, and IDO	Immunosuppression: inhibition of T cell proliferation and promotion of the transition of macrophages from the M1 to the M2 phenotype	Espagnolle et al^43^ Li et al^44^
Co-culture of HUVECs and MSCs using different distances	Distance between HUVECs and MSCs	MSC-EVs containing VEGF, FGF-2, and ITGA3	Angiogenesis and proliferation of endothelial cells	Piard et al^45^
Injured rat model injected with ADSCs	Cell activation	SDF-1	Migration of MSCs	Mazini et al^46^
NOD/SCID/IL-2Rγ^null^ mouse model	Cell migration	NAD^+^	Migration and proliferation of MSCs	Cavaliere et al^47^
Mouse model in the telogen stage of the hair cycle	Cell migration	PDGF-D	Migration and proliferation of MSCs	Hye Kim et al^48^
Mouse wound healing model	Cell migration	HGF	Migration of MSCs	Shi et al^49^
Mouse model with Foxf1 overexpression	Cell migration	ATX	Migration of MSCs	Cao et al^50^
Culture of hUCB-MSCs	Cell proliferation	PGE2	Improvement of the self-renewal capacity	Lee et al^51^
Culture of BMSCs	Cell proliferation	FGF-2	Proliferation of MSCs	Eom et al^52^
Culture of MSCs	Cell proliferation	bFGF	Proliferation of MSCs	Candini et al^53^
Mouse colorectal cancer model	Cell proliferation	TSG-6	Stemness of MSCs: affecting the cytoskeleton	Romano et al^54^
Culture of BMSCs	Cell proliferation	MMP-2	Stemness of MSCs: affecting ECM remodeling	Sassoli et al^55^
Rat model of femoral fracture	Proliferation stimulated by melatonin and osteogenic differentiation	Neuropeptide Y	Osteogenic differentiation of MSCs	Dong et al^56^
Culture of MSCs	Osteogenic differentiation	bFGF	Osteogenic differentiation	Candini et al^53^
Rat calvarial defect model	Osteogenic differentiation	IL-1α	Osteoinductive effects	Liu et al^59^
Culture of BMSCs *in vitro*	Osteogenic differentiation	IL-6	Osteogenic differentiation of BMSCs	Xie et al^60^
Culture of BMSCs on substrates with plant virus-modified nano-topography	Osteogenic differentiation	BMP-2	Osteogenic differentiation of BMSCs	Metavarayuth et al^61^
Culture of MSCs regulated by Angptl2 siRNA	Osteogenic differentiation	Angptl2	Regulation of bone metabolism	Takano et al^62^
Culture of BMSCs	Adipogenic differentiation	HGF	Adipogenic differentiation	Eom et al^52^
Mouse colorectal cancer model	Adipogenic differentiation	TSG-6	Adipogenic differentiation	Romano et al^54^
Culture of MSCs regulated by lncRNA ROA	Adipogenic differentiation	PTX3	Adipogenic differentiation	Pan et al^64^
Mouse model with high-fat diet	Adipogenic differentiation	MMP-2 and MMP-13	Adipogenic differentiation	Shih et al^65^
Culture of hUCB-MSCs treated with TSP2-targeting siRNA	Chondrogenic differentiation	TSP-2	Formation of cartilage	Jeong et al^66^
Culture of hMSCs	Chondrogenic differentiation	MMP-13	Chondrogenic differentiation	Salinas et al^67^
Culture of PMSCs	Muscle differentiation	IGF-2	Muscle differentiation	Aboalola et al^68^
Culture of MSCs	Cell senescence	miR-196a	Cell senescence	Candini et al^53^
Culture of cMSCs	Cell senescence	CCL2, CCL8, CX3CL1, HGF, GDF6, and MMP3	Sustain the senescence of cMSCs	Martini et al^69^
Culture of ADSCs	Cell senescence	IL-4, IP-10, PF4, Activin A, and DPP4	Attenuation of the angiogenic potential of ASCs	Ratushnyy et al^70^
Culture of MSCs	Cell senescence	IL-6	Induction and maintenance of aging of MSCs	Lehmann et al^71^
Mouse irradiation model	Cell senescence	IGFBP	Senescence of undamaged cells	Alessio et al^72^
MSCs treated with amphiregulin	Cell senescence	Amphiregulin	Cell proliferation and the EMT	von Joest et al^73^
Culture of MSCs	Cell autophagy	ANG	Proangiogenic activity	Lee et al^77^
Rat model of MI	Cell autophagy	EGF	Proangiogenic activity	Zhang et al^78^
PDLSCs treated with inflammatory cytokines	Cell autophagy	bFGF	Angiogenesis of PDLSCs	Wei et al^79^
Mouse full-thickness cutaneous wound model	Cell autophagy	VEGF	Angiogenesis of endothelial cells	An et al^80^
Culture of MSCs with rapamycin pretreatment	Cell autophagy	TGF-β1	Immunomodulation: inhibition of proliferation of CD4^+^ T cells	Gao et al^81^
Rabbit wound healing model	Cell apoptosis	TSG-6	Inhibition of hypertrophic scar formation	Liu et al^86^
Culture of hypox-visASCs from patients with metabolic syndrome	Cell apoptosis	IL-1β, TNF- α, HGF, and VEGF	Increase inflammation and inhibit inflammation and angiogenesis of endothelial cells	Oliva-Olivera et al^19^
Mouse MI model	Cell apoptosis	Apoptotic bodies	Angiogenesis of endothelial cells	Liu et al^87^
Mouse skin wound healing model	Cell apoptosis	Apoptotic bodies	Polarization of macrophages toward the M2 phenotype	Liu et al^88^

Abbreviations: ADSCs, adipose-derived stem cells; ANG, angiotensin; Angtl2, angiogenin-like protein 2; ATX, autotaxin; bFGF, basic fibroblast growth factor; BMSCs, bone marrow mesenchymal stem cells; cMSCs, cardiac mesenchymal stromal cells; DPP4, dipeptidyl peptidase 4; EGF, epidermal growth factor; FGF-2, fibroblast growth factor-2; Foxf1, Forkhead box protein F1; GDF, growth/differentiation factor; HGF, hepatocyte growth factor; Hsp90, heat shock protein 90; hUCB-MSCs, human umbilical cord blood mesenchymal stem cells; HUVECs, human umbilical vein endothelial cells; hypox-visASCs, visceral adipose derived mesenchymal stromal cells in hypoxia; IDO, indoleamine 2,3-dioxygenase 1; IGFBP, insulin-like growth factor-binding protein; IGTA3, integrin subunit alpha 3; MI, myocardial infarction; MMP, matrix metalloproteinase; NOD/SCID/IL-2Rγ^null^, nonobese diabetic/severe combined immunodeficient disease IL-2 receptor γ-null mice; PDGF, platelet-derived growth factor; PDLSCs, periodontal ligament stem cells; PGE2, phenyl glycidyl ether-2; PMSCs, placental mesenchymal stem cells; PTX3, pentraxin3; SDF-1, stromal cell-derived factor-1; TGF, transforming growth factor; TSG-6, tumor necrosis factor-stimulating gene 6; TSP-2, human thrombospondin-2; VEGF, vascular endothelial growth factor.

## The stem cell secretome

MSCs can secrete a series of biologically active molecules, called the secretome, which are usually classified as cytokines, chemokines, cell adhesion molecules, lipid mediators, interleukins (ILs), growth factors, hormones, extracellular vesicles (EVs), and other molecules. These factors are considered to be the protagonists^4^ in intercellular signal transduction, crosstalk between cells and the microenvironment, and tissue repair and regeneration, because they influence various biological processes such as angiogenesis, their anti-inflammatory, immunomodulatory,^5^^,^^6^ anti-fibrotic, and anti-tumor effects, and their stimulatory effects on cell migration, proliferation, and differentiation. The secretome of MSCs has great therapeutic potential in different disease models, while the identities and concentrations of these biologically active molecules may greatly differ depending on the microenvironment and biological behavior.^7^^,^^8^ Therefore, it is critical to understand the factors that affect the secretome of MSCs because this will allow the secretome to be modulated to elicit improved therapeutic effects.

## The stem cell secretome in different microenvironments

### Inflammatory microenvironment

Inflammation is an important pathological process. When inflammatory damage occurs, a local hypoxic environment containing inflammatory cytokines such as interferon (IFN)-γ and tumor necrosis factor (TNF)-α are created. MSCs can detect the inflammatory microenvironment and elicit anti-inflammatory and immunomodulatory effects via their paracrine functions to trigger regeneration. A large amount of evidence demonstrates that hypoxia and inflammatory cytokines are two important factors that affect the secretion profile of MSCs in an inflammatory environment. Hypoxia and inflammatory cytokines can recruit MSCs to areas of inflammatory injury, stimulate them to produce a variety of cytokines and chemokines, and then promote the repair and regeneration of damaged tissues.

Hypoxic preconditioning of MSCs can enhance their paracrine effects by stimulating them to produce a variety of bioactive factors. For instance, a study of inflammatory bowel disease reported that under hypoxia, MSCs produce a large amount of insulin-like growth factor (IGF)-2, which instructs maturing macrophages to perform oxidative phosphorylation and acquire anti-inflammatory properties.^9^ Gao et al found that after myocardial infarction (MI), serum exosomes produced at sites of ischemic and hypoxic injury deliver miR-1956 and activate paracrine proangiogenic vascular endothelial growth factor (VEGF) signaling in adipose-derived MSCs (ADSCs).^10^ Bernard et al showed that the protective paracrine effect of human MSCs (hMSCs) was partly dependent on the secretion of keratinocyte growth factor and hepatocyte growth factor (HGF), which prevented the accumulation of reactive oxygen species (ROS) and hypoxia-inducible factor (HIF)-1α, in rat models of alveolar injury.^11^ In addition, hypoxia can induce the expression of HIF-1α and increase the release of EVs, which transfer mitochondria, miRNAs, proteins, and other functional substances to receptor cells to suppress inflammation and induce tissue repair. Under hypoxia, MSCs secrete EVs overexpressing miR-30b-3p or miR-21-5p to inhibit apoptosis of alveolar epithelial cells and alleviate ischemia-reperfusion injury in the lungs.^12^^,^^13^ Similarly, in mouse full-thickness skin injury and bone fracture models, administration of hypoxic exosomes promoted angiogenesis, proliferation, and migration of endothelial cells more than administration of normoxic exosomes because hypoxic preconditioning enhanced production of miR-126 and miR-125b by activating HIF-1α.^14^^,^^15^ Rong et al found that HIF-1α induced hypoxic bone marrow MSCs (BMSCs) to release EVs, which promoted proliferation and migration and inhibited apoptosis of chondrocytes through the miR-216a-5p/JAK2/STAT3 signaling pathway in an osteoarthritis model.^16^ Furthermore, in the inflammatory microenvironment of acute respiratory distress syndrome, MSCs transfer mitochondria and Ang-1 mRNA to macrophages by releasing EVs to promote their transformation to the M2 phenotype, which is an anti-inflammatory and highly phagocytic phenotype.^17^^,^^18^ Therefore, hypoxia pretreatment is a potential method to maximize the therapeutic effect of MSCs on inflammation.

Inflammation is accompanied by activation of the immune system, and the inflammatory microenvironment is filled with many immune cells that secrete many inflammatory mediators, forming a complex cytokine network. These inflammatory factors can directly promote the paracrine effects of MSCs and thereby the repair process. IL-1β stimulation can increase the secretion of cytokines, such as IL-4, IL-10, IL-8, IL-13, fibroblast growth factor (FGF)-2, epidermal growth factor (EGF), VEGF-A, and monocyte chemoattractant protein (MCP)-1, by ADSCs.^19^ Moreover, the secretome of MSCs can be greatly enriched and amplified by treatment with IFN-γ, TNF-α, and especially VEGF-C, which promotes angiogenesis and accelerates skin wound healing.^20^ When MSCs were treated with TNF-α alone, secretion of IL-6 was increased and transformation of macrophages to the M2 phenotype was promoted.^21^ These results provide a new strategy to maximize the paracrine effects of MSCs by using inflammatory factors. In addition, hypoxia, an extracellular acidic environment, and inflammatory mediators in the inflammatory microenvironment also exist in the tumor microenvironment (TME), which can also affect the paracrine function of MSCs.

### Tumor microenvironment

The TME is an interactive cellular environment around tumors. Its main function is to establish cellular communication pathways that support tumorigenesis. Tumor cells can create a “tumor niche” through cell–cell contacts or paracrine effects, and change the functions of normal cells, such as the paracrine effects of MSCs. MSCs in the TME participate in ECM remodeling and the epithelial-mesenchymal transformation (EMT) by secreting many soluble factors, such as cytokines, chemokines, and growth factors, to mediate crosstalk between tumor cells and the ECM, and affect tumor development. In addition, hypoxia, an extracellular acidic environment, and inflammatory mediators are also present in the TME, which can also affect the paracrine functions of MSCs.

In the TME, MSCs enhance paracrine effects by contacting tumor cells and then crosstalk with other cells in the TME, which is involved in tumorigenesis and development, metastasis, dormancy, and immunoregulation of tumors. MSCs up-regulate the secreted level of transforming growth factor (TGF)-β and promote EMT of tumor cells through cell–cell contact.^22^ As early as 2007, studies confirmed that contact between MSCs and breast cancer cells (BCCs) in the breast cancer environment increased the secretion of CCL5 and SDF-1α and the metastatic potential of cancer cells.^23^ In addition, upon contact with tumor cells, MSCs can reversely transfer nucleic acids to these cells through exosomes to regulate tumor progression. Upon cell–cell contact, MSCs secrete EVs rich in lncRNAPVT1 to promote the proliferation, migration, and invasion of osteosarcoma cells.^24^ Similarly, MSC-derived EVs enriched with miR-130b-3p play an oncogenic role in lung cancer progression,^25^ and miR-23b-enriched exosomes secreted by BMSCs promote BCC dormancy in a metastatic niche.^26^ Interestingly, recent studies showed that MSCs can also inhibit tumor growth through contact-regulated paracrine effects. Pakravan et al showed that miR-100 is enriched in MSC-derived exosomes by cell–cell contact and its transfer to breast cancer-derived cells is associated with the down-regulation of VEGF to inhibit tumor development.^27^ In addition, BMSC-EV-derived let-7i inhibits lung cancer progression.^28^

Tumor cell-derived exosomes (TEX) are ubiquitous in the TME and are the main participants in intercellular crosstalk. They transmit information from tumor cells to other normal or malignant cells in the TME, including MSCs, and thereby affect their functions and phenotypes. Evidence suggests that lung tumor cell A549-derived exosomes can induce MSCs to acquire a pro-inflammatory phenotype, named P-MSCs, which exhibit significantly elevated secretion of IL-6, IL-8, and MCP-1 through the NF-κB-TLR signaling pathway.^29^ TEX produced by acute myelocytic leukemia (AML) cells induced widespread down-regulation of hematopoietic stem cell-supporting factors, such as CXCL12, KITL, IL-7, and IGF-1, in BMSCs and reduced their ability to support normal hematopoiesis.^30^ Cancer stem cell-derived EVs in renal cell carcinoma promoted persistent phenotypic changes of MSCs characterized by increased expression of genes associated with cell migration, matrix remodeling, angiogenesis, and tumor growth such as those encoding CXCR4, COL4A3, IL-8, osteopontin, and myeloperoxidase.^31^ Furthermore, findings suggest that EVs secreted by both highly malignant osteosarcoma cells and human cholangiocarcinoma cells selectively incorporate a membrane-associated form of TGF-β, which induces pro-inflammatory IL-6 production by MSCs to enhance tumor progression.^32^^,^^33^

Hypoxia and inflammatory factors also exist in the TME, which affect the paracrine function of MSCs. IFN-γ and TNF-α in the TME stimulate MSCs to secrete TGF-β1, which induces the EMT in melanoma, breast cancer, hepatocellular carcinoma, and pancreatic adenocarcinoma cells, and increases the resistance of tumor cells to apoptosis, enhancing the tumor invasive ability.^34^ Hypoxia can increase the secretion of IL-1β by hMSCs in the TME, which inhibits T cell proliferation to induce inflammation and immune escape.^35^

### Biological microenvironment

MSCs are exposed to a variety of mechanical forces in the physiological environment. They are also mechanical force-sensitive stem cells that can respond to different mechanical forces, such as mechanical tension, compression, and shear stress. Different types of mechanical forces may have different effects on the fate and function of MSCs, including their paracrine function and immunoregulatory ability. Some studies explored the effect of laminar shear stress (LS) on the paracrine function of MSCs. Activation of the Wnt/β-catenin signaling pathway in MSCs stimulated by LS increased the secretion of proteins related to migration, proliferation, and angiogenesis of endothelial cells, and improved the resistance of these cells to oxidative stress.^36^ Under cyclic compression, expression of bone morphogenetic protein (BMP) is up-regulated in MSCs, which promotes their osteogenic differentiation.^37^ Meanwhile, some findings have demonstrated that matrix stiffness can affect the paracrine function of MSCs. An increase in the ECM stiffness promotes the paracrine function of MSCs.^38^ For example, secretion of VEGF depends on the stiffness of the matrix, and secretion was maximal when MSCs were seeded on hydrogel matrices with a stiffness of 5.0 kPa.^39^

In addition, ECM molecules can support the paracrine function of MSCs under physiological conditions. Among them, cadherin is a force sensor that can activate cytoskeleton remodeling and signal transduction in response to changes in intercellular tension. MSCs cultured on the full N-cadherin extracellular domain (EC1-5) exhibited stiffness-dependent changes and significantly higher secretion of VEGF and IGF-1.^40^ In a MI model, laminin can enhance secretion of human growth regulatory oncogene protein (GRO)-α/IL-8 and HGF through the JNK and PI3K/AKT signaling pathways, respectively, in placental-derived stem cells, which inhibits ROS production and cardiomyocyte apoptosis.^41^

In addition to changes in the microenvironment, a series of changes in the biological behavior of MSCs occur when the body is damaged. MSCs first migrate from the bone marrow or another tissue niche to the peripheral circulation and are then recruited to the injured or ischemic site.^42^ This is followed by the formation of cell–cell contacts, proliferation, differentiation, and apoptosis to promote tissue repair. These behaviors also stimulate the paracrine effect of MSCs and change the composition of their secretome to facilitate regeneration of the injured site.

## The secretome of stem cells with different biological behaviors

### Cell–cell contact and intercellular distance

An increasing body of evidence shows that the immunomodulatory characteristics of MSCs depend on their paracrine factors, and direct contact between cells plays a substantial role in this process. The interaction between MSCs and M1 macrophages up-regulate the expression of CD54, CD200, phenyl glycidyl ether-2 (PGE2), tumor necrosis factor-stimulating gene 6 protein (TSG-6), and IDO in MSCs, and thereby increase their immunosuppressive capacities by inhibiting T cell proliferation.^43^ At the same time, up-regulation of TSG-6 and CD200 can reversely mediate cell–cell contact between MSCs and M1 macrophages, promoting the transition of macrophages from the M1 to the M2 phenotype to induce immune tolerance.^44^

Moreover, when there is no cell–cell contact, the distance between cells affects the paracrine effects of MSCs. EVs are mainly involved in intercellular communication when cells are far apart, while paracrine signal transduction via soluble proteins plays a dominant role when cells are closer together. Piard et al studied the effect of the distance between endothelial cells and MSCs on paracrine functions and found that up-regulation of VEGF, FGF-2, and ITGA3 (integrins) in EVs produced by MSCs was increased in the group with the largest intercellular distance (> 400 μm). Regulation of the distance between cells generates different paracrine gradients and stimulates crosstalk between human umbilical vein endothelial cells and MSCs.^45^ These results suggest that the secretome of stem cells can be regulated by controlling the distance between cells.

### Cell homing and migration

The homing and migration of MSCs are mainly divided into four steps: tethering and rolling, activation, arrest, and transmigration. The expression of different molecules is up-regulated at different stages to facilitate the entire process. The first step is mainly induced by the expression of selectin in endothelial cells, and the activation step mainly involves chemokines and their receptors. In the arrest step, the integrin expressed by MSCs binds to cell adhesion molecules, and matrix metalloproteinases (MMPs) play a major role in the final stage.

Chemokines secreted by MSCs play a decisive role in cell activation. The stromal cell-derived factor (SDF)-1/CXCR4 axis plays a vital role in maintaining the function and development of other precursor cells in tissues. SDF-1 is overexpressed during activation of MSCs and enhances migration of these cells through CXCR4.^46^ For successful transmigration, MSCs secrete increasing amounts of MMPs to degrade the basement membrane. Furthermore, NAD^+47^ and growth factors such as platelet-derived growth factor (PDGF)-D^48^ and HGF,^49^ which promote cell migration and proliferation, are also highly expressed during the migration of MSCs. Recent studies also found that resident MSCs in the lungs can regulate the expression of extracellular lysophosphatidic acid (LPA) by secreting the autocrine motility-stimulating factor autotaxin during migration to the injured site, and LPA is the key inducer of MSC migration and can further induce directional migration.^50^

### Cell proliferation

The self-renewal ability of MSCs is central to their therapeutic effects. After migrating to the damaged area, MSCs can promote tissue healing by proliferating and eliciting paracrine effects. The proliferation of MSCs enhances their paracrine effects at injured sites. During the proliferation of MSCs, expression of PGE2 is up-regulated, which helps to maintain the self-renewal capacity through EP2.^51^ FGF-2 and basic FGF (bFGF), which are vital regulators of stem cell proliferation, are up-regulated during proliferation of MSCs and target the AKT/ERK pathway^52^ and *HOXB7* gene,^53^ respectively, to induce continuous proliferation. In addition, TSG-6,^54^ sphingosine-1-phosphate, and MMP-2^55^ are up-regulated during the proliferation of MSCs, which maintains the stemness of MSCs by affecting the remodeling of the cytoskeleton and surrounding ECM, while MMP-2 is down-regulated under hypoxia. It was recently demonstrated that neuropeptide Y (NPY) can be expressed in non-neuronal cells such as osteoblasts and BMSCs in the bone marrow microenvironment,^56^ and its expression increases upon the melatonin-stimulated proliferation of BMSCs.

### Cell differentiation

Proliferation and differentiation are usually regarded as two sides of the same coin. A cell will differentiate once it stops proliferating. During differentiation, MSCs secrete Abi3bp, a novel ECM protein that promotes the switch from proliferation to differentiation in MSCs by inhibiting ERK1/2 and cyclin-D1.^57^ Thereafter, MSCs can undergo osteogenic, chondrogenic, and adipogenic differentiation, and secrete a variety of bioactive substances to regulate the entire differentiation process. Much evidence shows that there is an inverse relationship between the secretomes of MSCs when they differentiate into osteoblasts or adipocytes.^47^ Kim et al identified the proteins secreted by BMSCs during osteogenesis and found that 177 proteins were up-regulated,^58^ including IL-1α,^59^ IL-6,^60^ BMP-2,^61^ VEGF-A, and bFGF,^53^ which induced synthesis of downstream osteogenesis-related proteins, such as collagen and osteopontin, and promoted bone formation. Takano et al found that angiogenin-like protein 2 (Angptl2), a positive regulator of cell differentiation, is highly expressed in MSCs and osteoblasts, and regulates bone metabolism.^62^ Recent studies found that melatonin promotes the healing of femoral fractures in a rat model and that MSCs can also promote the secretion of NPY during osteogenic differentiation.^63^ During adipogenic differentiation of MSCs, secretion of most of the above-mentioned bioactive factors was down-regulated, but the entire differentiation process was maintained due to the up-regulation of PTX3,^64^ HGF,^52^ TSG-6,^54^ MMP-2, and MMP-13.^65^ Moreover, during chondrogenic differentiation of MSCs, secretion of TSP-2,^66^ which promotes the formation of cartilage through the Notch signaling pathway, and MMP-13,^67^ which determines the fate of MSCs by regulating integrins, increased. Furthermore, there is evidence that the capacity of placental MSCs to synthesize IGF-2 increases during muscle differentiation.^68^

### Cell senescence

Aging is the main risk factor for chronic diseases, and the development of age-related diseases depends on the induction of cell senescence. Cell senescence can lead to resistance to apoptosis and expression of cell cycle inhibitors, and produce the senescence-associated secretory phenotype (SASP). The SASP includes a variety of bioactive factors, such as growth factors, pro-inflammatory cytokines, chemokines, and proteases, which increase the sensitivity of neighboring normal cells to the paracrine activities of aging cells and thereby enhance the entire aging process. For example, secretion of chemokines (CCL2, CCL8, and CX3CL1), growth factors (HGF and GDF6), a protease (MMP3), and periostin is increased in aging myocardial MSCs.^69^ By contrast, anti-angiogenic factors (*e.g.*, IL-4, IP-10, PF4, Activin A, and dipeptidyl peptidase 4 (DPP4)) were up-regulated and angiogenic factors (IGF-1, MMP1, TGF-B3, PDGFRB, and PGF) were down-regulated in aging ADSCs.^70^ In addition, IL-6 in the huge SASP factor network is considered to play a key role in the induction and maintenance of aging, which can elicit a positive feedback effect to accelerate cell senescence.^71^ Furthermore, aging cells secrete IGFBP, a genotoxic stress mediator, which promotes the senescence of undamaged cells when released into the bloodstream.^72^ The latest research shows that aging MSCs can secrete amphiregulin, which accelerates cell proliferation and the EMT through EGF receptor signal transduction and facilitates cellular plasticity to promote reprogramming and tissue repair.^73^

The expression of some miRNAs is also regulated in senescent MSCs. For instance, miR-196a is up-regulated to affect cell proliferation and promote cell senescence.^53^ Meanwhile, miR-146a^74^ and miR-10a^75^ are down-regulated and the angiogenic ability of exosomes is inhibited.

### Cell autophagy and apoptosis

Autophagy is a highly conserved catabolic process induced by various cellular stresses that protect cells. When energy or nutrition is lacking, autophagy is induced in MSCs in response to various cytotoxic injuries to delay aging and avoid apoptosis.^76^ In addition, cells utilize autophagy to secrete cytoplasmic components, which regulate a variety of pathological processes to promote the regeneration of injured sites. Autophagy drives the secretion of angiogenic factors such as ANG,^77^ EGF,^78^ bFGF,^79^ and VEGF^80^ by MSCs, which underlies the repair role of these cells in a variety of injury models. Furthermore, rapamycin pretreatment increases autophagy in MSCs, enhances secretion of TGF-β1, and inhibits the proliferation of CD4^+^ T cells to elicit immunomodulatory effects.^81^ Marcelin et al found that inhibition of autophagy in AMSCs remodels the balance between TGFs and BMPs, which reduces gene expression of ECM molecules and weakens the fibrotic response of adipose tissue to a high-fat diet.^82^

Autophagy and apoptosis usually occur in the same cell, and autophagy mainly precedes apoptosis. This is because stress usually stimulates autophagy, especially if it is below the level that causes cell death. However, when the stress exceeds the critical duration or intensity threshold, apoptosis is activated. Apoptosis is a form of programmed cell death, which helps to eliminate aging and damaged cells, and plays a vital role in maintaining physiological homeostasis. During apoptosis, MSCs secrete a variety of soluble proteins and apoptotic EVs (apoEVs).^83^^,^^84^ Therapeutically applied MSCs undergo apoptosis and release apoEVs, which facilitates their therapeutic effects.^85^ hMSCs can effectively inhibit the formation of hypertrophic scars by promoting the secretion of TSG-6 during apoptosis.^86^ After apoptosis was induced by TNF-α, the levels of pro-inflammatory, anti-inflammatory, and angiogenic cytokines secreted by MSCs, such as IL-1β, TNF-α, HGF, and VEGF, increased, promoting repair of damaged sites.^19^ After MSCs were implanted into the MI area of mice, they underwent apoptosis and secreted many apoptotic bodies (ABs), which enhanced the angiogenesis of endothelial cells in the transplanted area.^87^ Moreover, ABs derived from MSCs promoted cutaneous wound healing by triggering the polarization of macrophages toward the M2 phenotype.^88^

### Clinical applications and prospects

As the therapeutic potential of the stem cell secretome has gradually been discovered, an increasing number of researchers have investigated employing paracrine effects as the main mechanism of MSC-based therapy and attempted to modulate the secretome by pretreating MSCs. For instance, the secretion profile and therapeutic activity of MSCs can be regulated by adjusting the growth microenvironment of MSCs, using bioactive agents, or employing biomaterials.

The most common pretreatment method is to cultivate MSCs in hypoxia (0.1% O_2_) and collect MSC-CM. Hypoxia can promote the proliferation and migration of MSCs, and increase the expression of cytokines such as VEGF, IL-6, IL-15, and IL-1β.^89^ In addition, supplementation of biologically active agents is also a common means to regulate MSCs. For example, the direct addition of TGF-β1 to MSCs increases the production of several factors involved in bone remodeling, including CXCL9, CCL26, and osteopontin.^90^ Pretreatment with deferrioxamine increases secretion of angiogenic growth factors such as VEGF by MSCs and thereby improves fat graft retention.^91^ Pretreatment with valproic acid increases the expression of IL-10 in MSCs and enhances their anti-inflammatory activity.^92^ With the development of material science, scaffolds, which serve as a matrix for cell attachment and growth, have become an important part of tissue engineering. A special microenvironment can be created by modulating the microstructure, surface morphology, and mechanical properties of scaffolds, and the interaction between cells and materials can affect cellular behaviors. The fibrous topography of scaffolds is a key property that modulates the paracrine function of cells. Directional fibers can enhance the expression of PGE2, iNOS, and HGF, and induce an anti-inflammatory response in macrophages.^93^ Recent studies found that 3D culture, such as that using scaffolds, hydrogels, and spheres, increases secretion of cell-interacting proteins such as β-catenin and integrin-β1 as well as VEGF,^94^ HGF, and IL-10^95^ by MSCs compared with traditional 2D adherent culture. In addition, when MSCs were cultured on polyacrylamide hydrogels with different levels of stiffness, secretion of VEGF and IGF increased as stiffness increased.^96^

Clinical trials have been performed to treat cardiovascular and degenerative diseases using single cytokines, but the results were unsatisfactory. This suggests that a variety of bioactive substances should be used to achieve the best clinical results. Therefore, the MSC secretome and its therapeutic active components may be the best choice for cell-free therapy. Meanwhile, pretreatment can change the secretion profile of MSCs, and MSC-CM can be mass-produced. The MSC secretome has been utilized in a variety of animal models of inflammation and injury and achieved promising therapeutic results. For example, after induction of colitis in mice, intraperitoneal injection of MSC-CM significantly increases the levels of IL-10 and TGF-β in mesenteric lymph nodes and the spleen, elicits anti-inflammatory effects, and reduces colitis and mortality.^97^ Similarly, in a model of inflammatory arthritis, intra-articular injection of mouse MSC-CM reduces cartilage damage due to high expression of IL-10.^98^ In a mouse model of hepatic fibrosis, the number of activated hepatic stellate cells expressing α-SMA and the area of hepatic fibrosis decrease after injection of the MSC secretome.^99^ In a rat model of a skull defect, MSC-EVs promote bone regeneration and angiogenesis in the early stage.^100^ Although the results obtained using animal models support the utility of the MSC secretome, much work needs to be performed for these results to be translated into clinical practice. For example, the parameters for the preconditioning of MSCs need to be determined, the storage and delivery methods of the secretome need to be investigated, and the stability and safety of MSC-CM need to be evaluated. This is essential for further application of the MSC secretome for clinical treatment.

## Conclusions

MSCs are powerful bioactive agents for treating various diseases due to their paracrine actions. Investigations of the secretome of MSCs under various conditions should improve understanding of the immunoregulatory function and repair capability of these cells. The microenvironment and biological behavior of MSCs affect their paracrine activity. With the development of bioengineering and elucidation of the factors that affect the paracrine activity of MSCs, pretreatment of MSCs to regulate their secretion profile is a new approach to preparing the MSC secretome for cell-free therapy and opens up a new avenue for regenerative medicine.

## Author contributions

All authors contributed to researching the data for the work and writing the manuscript. ZX drafted the main text and tables. YL and YL supervised the work and provided comments and additional scientific information. ZX also reviewed and revised the text. All authors read and approved the final version of the manuscript to be published.

## Conflict of interests

The authors declare that there is no conflict of interests.

## Funding

This work was supported by the Natural Science Foundation of Guangdong Province of China (No. 2021A1515011623), the Administrator Foundation of Nanfang Hospital (China) (No. 2019B021, 2020Z004), and the National Nature Science Foundation of China (No. 81971852).
